# Parents’ Perception of Pediatricians on Social Media: The Emerging Role of Pediatric Health Communicator

**DOI:** 10.3390/children13070923

**Published:** 2026-07-13

**Authors:** Angelica Dessì, Elena Esposito, Ulrica Pani, Roberta Scanu, Vassilios Fanos, Alice Bosco

**Affiliations:** Neonatal Intensive Care Unit, Department of Surgical Sciences, University of Cagliari, AOU Cagliari, 09124 Cagliari, Italy

**Keywords:** social media, pediatric health communicator, parental perception

## Abstract

Background/Objectives: Social media is an increasingly important source of pediatric health information for parents. In particular, the growing presence of pediatricians on social networks opens up new scenarios for adapting to the communication models of the younger generations, supporting information, prevention and parental support, but also with critical issues related to integration with the traditional clinical relationship. This study aimed to evaluate the use of social media for pediatric health information, the perceived usefulness of content shared by pediatricians, its impact on parental behavior and the degree of integration of this information into dialogue with the treating pediatrician. Methods: A cross-sectional observational study was conducted using an online questionnaire aimed at 453 parents of minors. The questionnaire investigated socio-demographic characteristics, frequency of following pediatricians on social media, perceived usefulness of online health information, impact on parenting behaviors, discussion of content with the treating physician, and perception of risks and the need for regulation. Associations between categorical variables were evaluated using Fisher’s exact test and multivariable logistic regression. Results: Most participants follow pediatricians on social media (74.2%) and rate online health information as very useful (81.7%). Almost two-thirds reported changing at least one health-related behavior following exposure to pediatric content on social media (65%). Greater frequency of following pediatricians was significantly associated with both higher perceived usefulness and behavioral change (*p* < 0.0001). Perceived usefulness emerged as the strongest independent predictor of behavioral change (OR 9.967; *p* < 0.001). Conclusions: Pediatric communication on social media appears to be widely used, perceived as useful and associated with parental behavior. However, such content is poorly integrated into clinical dialogue, mainly taking the form of a one-way flow of information. The results highlight the need to promote more participatory communication models and to recognize pediatricians active on social media as a distinct professional category of digital health communicators.

## 1. Introduction

In 2025, the global number of social media users has reached more than 5 billion [1]. In Western Europe, social media penetration is among the highest globally [2], with more than 41 million active users in Italy [3]. These circumstances have necessitated the evolution of communication strategies, including in the scientific and academic fields. This is due to the fact that social media has changed the nature and speed of interaction between people, providing a new model of sharing and discussion that is no longer static and unidirectional, but participatory and immediate (real time). Adaptation to this new model is therefore now necessary in the scientific field as well [4,5]. Scientific literature has in fact highlighted the presence of a new communication ecosystem in the healthcare field, born precisely from the transition from traditional (editorial and institutional) vertical communication, where the message starts from the expert and reaches the public without response or dialogue, to a new and interactive form of dissemination. This type of participatory communication, which characterizes the world of social media, is rooted in audience interaction through messages and comments, but also through sharing, which transforms users into an active part of the communication process [5]. The advantages of this new approach to communication are numerous, starting with increased trust, sense of closeness, and greater understanding of health messages, to increased visibility and engagement, allowing the reach of health communication to be amplified and a much wider audience to be reached than through traditional channels [5]. Inevitably, there are also several disadvantages, including the risk of misinformation and poor content quality, mainly due to a lack of editorial control, which facilitates the spread of fake news, but also due to platform algorithms that prioritize engagement over accuracy, thus favoring sensationalist or polarizing content [5,6,7,8,9]. Added to this is the lack of tools to verify the authenticity and competence of those providing the message [5,6,7,8,9], which is associated with the fact that perceived credibility does not always depend on actual competence but on their ability to create emotional relationships and a sense of closeness with the audience [7]. These dynamics are amplified through the mechanism of algorithmic personalization, responsible for the creation of information bubbles, i.e., closed information environments where users only see content consistent with their beliefs. This condition reinforces pre-existing beliefs, sometimes undermining trust in health institutions [8,9]. In the context of personal use of social media, the risks of self-promotion, loss of professional boundaries, and personal overexposure should not be overlooked [10].

However, it is precisely the current inevitable process of digital transformation and, at the same time, the associated risks that require scientific societies to move beyond traditional editorial and institutional channels. It is now essential for them to take an active role in social media, participating directly in the construction of reliable, accessible health information that is consistent with the principles of scientific communication, especially in the pediatric field where communication responsibility is amplified because every message can influence, albeit indirectly, the health of minors and the choices of families. In this regard, the American Academy of Pediatrics (AAP) has long recognized social media as an educational and advocacy tool for both professionals and families, facilitating access to information and early identification of risky behaviors [10]. The North American Society of Pediatric Gastroenterology, Hepatology, and Nutrition (NASPGHAN) has also published a position paper inviting scientific societies and individual professionals to actively participate in digital communication, not only to combat misinformation but also to promote patient advocacy, the dissemination of educational content, and the dissemination of quality research. In addition, the society proposes both specific training for healthcare professionals in social communication and the recognition of online scientific dissemination as an activity worthy of academic and professional evaluation [11]. At the national level, the Italian Society of Pediatrics (SIP) has experimented with a concrete model through the creation of a group of pediatric influencers, pediatricians trained in digital communication tasked with disseminating validated messages and countering online misinformation, offering a virtuous example of integration between professional communication and digital languages [5]. Nevertheless, these documents often remain generic and non-binding, limiting themselves to principles of professionalism and caution without providing uniform operational guidelines regulating the boundaries between scientific communication and self-promotion, legal responsibility for informative content, and the recognition of dissemination as part of professional activity [6]. Furthermore, some studies have shown that, in practice, scientific communication remains predominantly unidirectional and expert-centered, more oriented toward the dissemination of information than toward dialogue with the public [12,13], an approach that proved unsuccessful during the COVID-19 pandemic and was responsible for low trust and reduced effectiveness of health messages [14].

Conversely, in pediatrics, where parents are the indirect recipients of health information, there is already ample scientific evidence to support the idea that online content influences parental choices regarding breastfeeding, nutrition, and vaccinations, often based on the perceived reliability of the source rather than the scientific quality of the message [15,16,17,18,19]. This is particularly relevant given that the broader digital information landscape, encompassing parenting blogs, online forums, and peer-led social media groups, is characterized by a predominance of non-professional content of highly variable quality, with a greater prevalence of misinformation than of accurate, evidence-based guidance [17,20,21,22]. Understanding how professionally qualified pediatricians, who currently represent less than 3% of all content creators in this space [6], can communicate effectively within this ecosystem therefore becomes a research priority. However, to date, few studies have specifically examined how parents perceive and evaluate health information shared by pediatricians on social media, and whether this professional form of digital communication is capable of influencing parental health behaviors and integrating into the clinical relationship. The present study addresses this gap by providing empirical evidence on how Italian parents perceive and interact with pediatric health communication on social media, with a specific focus on professionally qualified pediatricians as a distinct category of healthcare professional active on social media, and on the extent to which such communication translates into behavioral change and integrates into the clinical relationship.

## 2. Materials and Methods

### 2.1. Study Design

A cross-sectional observational study was conducted to explore parental perceptions of pediatric communication through social media. Data collection took place in June 2025 using a structured online questionnaire, administered anonymously and voluntarily. The link to the questionnaire was posted on the Instagram pages of some of the authors and shared via WhatsApp groups of parents belonging to nursery schools, primary schools, and secondary schools. This method of dissemination aimed to include parents with different levels of digital familiarity, broadening the variety of the sample compared to recruitment exclusively through social networks.

### 2.2. Participants and Recruitment

A total of 453 parents, all residing in Italy, participated. The inclusion criteria were parents of legal age with at least one child between the ages of 0 and 14, together with the completion of question 1 regarding the reading and understanding of the information on the study and informed consent to participate, while the exclusion criteria included incomplete questionnaires. Participation was voluntary and anonymous, with no incentives. No formal sample size calculation was performed, as the study was designed as an exploratory cross-sectional survey.

### 2.3. Data Collection Instrument

A structured questionnaire was used, developed on the basis of international literature on health communication and the use of social media in healthcare, with particular reference to studies analyzing the evolution of communication models in pediatrics: specifically, parents’ trust in digital sources and the role of healthcare professionals as disseminators [4,5,6,7,10,15,16,18]. The tool, distributed in digital format, included 17 items organized into four main thematic areas, with closed-ended (dichotomous or polychotomous) questions, multiple-choice questions, and three- or five-point Likert scale questions.

To minimize the risk of unintentional duplicate submissions, the platform was configured to hide the option to submit another response upon questionnaire completion. While this measure does not constitute a technical block against deliberate re-entry, it represents a platform-level control consistent with the anonymous design of the study.

The areas of investigation were as follows:

1. Socio-demographic data (4 items): these included the parent’s age, educational qualifications, number of children, and age group of the child in question.

2. General use of social media and perception of pediatric communication (9 items): this section explored methods, content, and perceptions related to the use of online pediatric information.

3. Impact on parenting behaviors (2 items): this section investigated whether and in what areas social media content had influenced parents’ decisions or behaviors.

4. Perception of risks and opinions on regulation (2 items): This section explored perceptions of the potential negative effects of pediatricians’ presence on social media and the need for specific rules for online disclosure.

Responses were collected using 3- or 5-point Likert scales, dichotomous items (yes/no) or polychotomous items (yes/no/do not remember), and multiple-choice questions with predefined answers.

Table 1 summarizes the structure of the questionnaire and the type of responses for each section.

### 2.4. Statistical Analysis

Data were collected using Microsoft Forms, managed in Microsoft Excel, analyzed using GraphPad Prism (version 10.6.1; GraphPad Software, San Diego, CA, USA) and R software (version 4.4.1; R Foundation for Statistical Computing, Vienna, Austria). Associations between categorical variables were evaluated using contingency tables and Fisher’s exact test, with the significance level set at *p* < 0.05. A multivariable logistic regression model was built using the enter method, including four predictors simultaneously: frequency of following pediatricians on social media, perceived usefulness of online health information, parental educational level, and parental age. In the logistic regression model, behavioral change was coded as a binary outcome (yes = 1, no = 0); participants who responded “I do not remember” were excluded from this analysis. Frequency of following pediatricians and perceived usefulness were treated as ordinal numeric variables; parental age was dichotomized (≤35 vs. >35 years) and educational level was treated as an ordinal variable.

### 2.5. Ethical Considerations

This study was conducted in accordance with EU Regulation 2016/679 (GDPR) and applicable national regulations governing observational research with anonymous data. The research protocol was reviewed by the Data Protection Officer (DPO) and the Privacy Office of the University of Cagliari, which determined that ethics committee approval was not required, as the study involved an anonymous online questionnaire with no collection of sensitive personal data and no clinical intervention. This determination was communicated via institutional correspondence dated 5 May 2025. Informed consent was obtained from all participants prior to questionnaire completion: the first item of the questionnaire required participants to explicitly confirm that they had read and understood the purposes and methods of data collection and that they consented to participate; completion of this item was mandatory, and participants who did not provide consent were unable to proceed. Participation was entirely voluntary and anonymous, and no personal identifying information was collected at any stage of the study.

## 3. Results

### 3.1. Survey Completion Time and Participants’ Sociodemographic Characteristics

The average time taken to complete the questionnaire was 3 min and 57 s. A total of 453 parents participated, all of whom stated that they had read and understood the purposes and methods of data collection and provided their informed consent.

In terms of sociodemographic characteristics, the age of the parents was mainly concentrated in the adult age groups: no participant was under 20 years of age; 4 (0.9%) were between 20 and 25 years old, 43 (9.5%) between 26 and 30 years old, 131 (28.9%) between 31 and 35 years old, and 275 (60.7%) over 35 years old.

The level of education shows a distribution oriented towards medium-high qualifications: 29 parents (6.4%) have a middle school diploma, 162 (35.8%) have a high school diploma, and 262 (57.8%) have a university degree.

In terms of family composition, 274 participants (60.5%) have one child, 158 (34.9%) have two, and 21 (4.6%) have three or more.

### 3.2. Engagement with Pediatric Content on Social Media and Perceived Usefulness of Information

**Figure 1:**
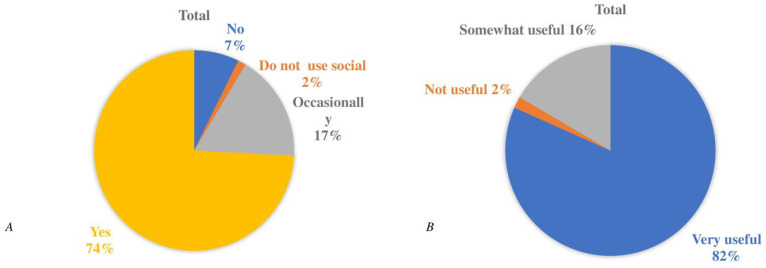
Panels (**A**,**B**): summary of the main descriptive findings: (**A**) parents’ interest in following pediatricians on social media; (**B**) the perceived usefulness of medical information found online.

Interest in pediatric content on social media is high (Figure 1A). In particular, most parents report frequently following one or more pediatricians, some do so only occasionally, while a minority do not follow any pediatrician and a small proportion do not use social media at all.

Medical information found on social media is considered very useful by more than 80% of participants, not very useful by 16.6%, and not useful at all by only 1.8% (Figure 1B).

**Figure 2:**
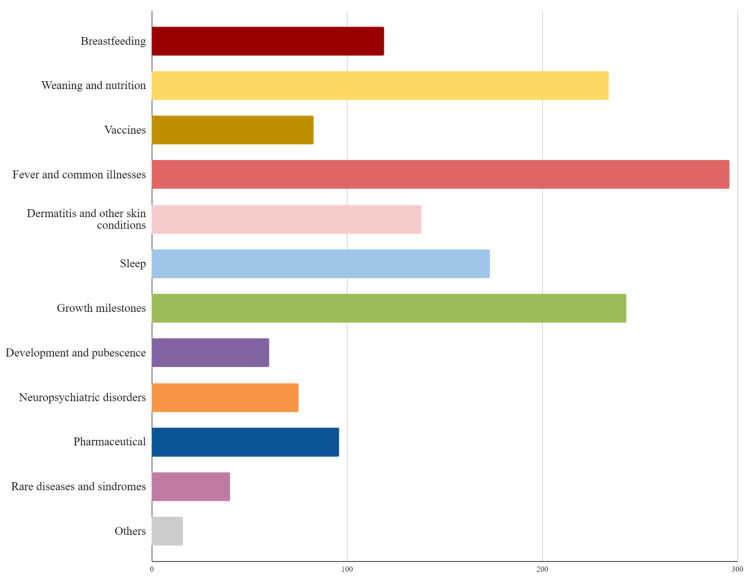
Frequency distribution of pediatric health topics searched online by parents (*n* = 453), reported as number of selections (multiple-response question).

With regard to the topics most frequently searched for online (multiple-choice question), the most recurring themes are: fever and common illnesses (296 selections), stages of growth (243), weaning and nutrition (234), sleep (173), dermatitis and skin problems (138), breastfeeding (119), medications (96), vaccinations (83), neuropsychiatric disorders (75), development and puberty (60), rare diseases and syndromes (40), and other (16) (Figure 2).

**Figure 3:**
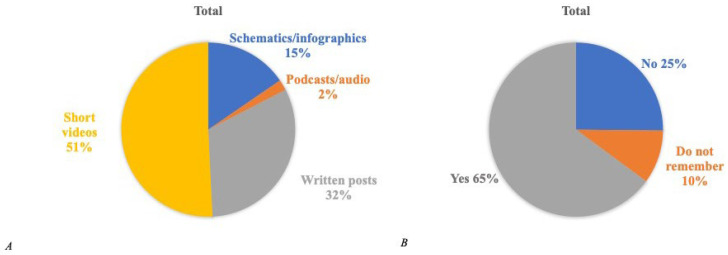
Panels (**A**,**B**). Summary of the main descriptive findings: (**A**) preferred modalities for consuming pediatric health content on social media; (**B**) percentage of parents reporting behavioral changes after exposure to pediatric health content on social media.

As for the preferred methods of content consumption, just over half of parents prefer short videos, followed by written posts and schematic images or infographics. A small minority prefer podcasts (Figure 3A).

A central aspect of the survey concerns the impact of informative content: more than 60% of parents say they have changed at least one behavior or decision related to their child’s health thanks to content shared by pediatricians on social media, while a quarter of the sample report no changes and less than 10% do not remember (Figure 3B).

Among those who changed their behavior (multiple-response question), the most frequently affected areas were: weaning/feeding (179 selections), fever and common illnesses (136), sleep (102), breastfeeding (78), vaccinations or medications (30), and other (17).

### 3.3. Discussion of Social Media Content with the Pediatrician

**Figure 4:**
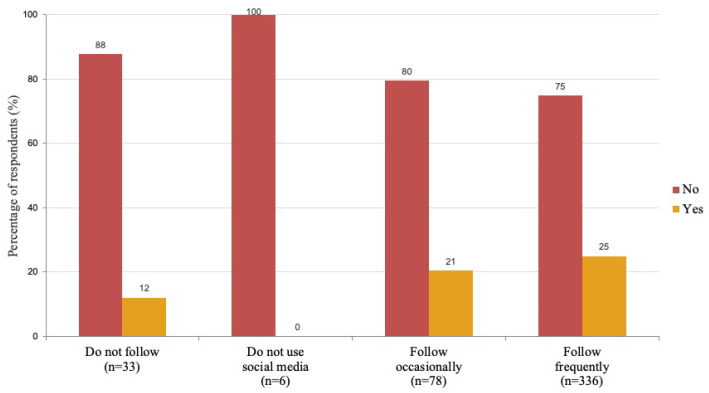
Distribution of parents who discussed pediatric social media content with their primary care physician (yes or no), stratified by frequency of following pediatricians on social media (453).

Only 22.9% of parents report having discussed pediatric content found on social media with their doctor, while 77.1% have never discussed it. Among those who did discuss it, 47.1% reported a positive or interested attitude on the part of the doctor, 24% reported a doubtful or cautious attitude, 16.3% reported being advised not to rely on online sources, and 12.5% reported an annoyed or uncooperative reaction. Figure 4 shows the distribution (number) of “yes” and “no” responses for each level of following on social media (I do not use social media; I do not follow pediatricians; I follow them occasionally; I follow them often). The graph shows that the proportion of parents who discuss such content with their doctor remains limited in all groups, regardless of their level of exposure to online pediatric content.

### 3.4. Perceived Impact, Risks and Regulation of Pediatricians’ Communication on Social Media

**Figure 5:**
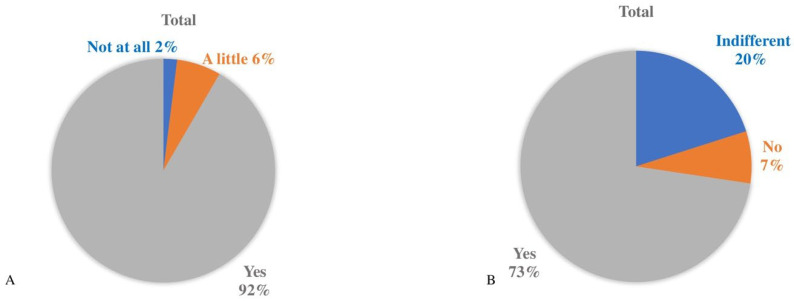
(Panels (**A**,**B**)). Summary of parents’ evaluations of pediatric communication on social media: (**A**) overall perceived usefulness of pediatricians’ communication for improving information, prevention and parental support; and (**B**) parents’ views on whether specific regulation would improve the safety of pediatric health information online.

Trust in online content is influenced by personal knowledge of the professional: 68.7% of parents say they would trust more if they knew the pediatrician who authored the content personally, while only 7.1% respond negatively and 24.3% say they are indifferent. The sharing of personal content by the pediatrician (e.g., daily life, family) has a moderate impact on trust, with an average score of 3.11 out of 5. A very high proportion of the sample (91.6%) believes that pediatricians’ communication on social media can improve information, prevention, and support for parents; 6.4% of parents consider it not very useful and only 2% consider it not useful at all (Figure 5A).

Among the perceived risks (multiple-choice question), the following emerge: replacement of medical visits with online consultations (229 selections; 50.6%), risk of misunderstandings (201 selections; 44.4%), confusion due to the presence of discordant opinions among pediatricians (197 selections; 43.5%), and dissemination of overly generic or non-personalized information (169 selections; 37.3%). Other risks reported include conflict of interest (137 selections, 30.2%), trivialization of issues (111 selections, 24.5%), media overexposure of the professional (49 selections, 10.8%), and other (9 selections, 2%). Only 11.9% of participants perceive no risk (percentages calculated on the total number of participants). The perception of risks appears to be distributed similarly across the three levels of education considered, with comparable percentages of parents reporting no risk, suggesting no significant differences between the groups.

Finally, 72.6% of participants believe that specific regulation would improve the safety of pediatric information online, while only 7.3% respond negatively and 20.1% remain indifferent (Figure 5B).

### 3.5. Inferential Analysis and Multivariable Logistic Regression

#### 3.5.1. Bivariate Inferential Analysis

To further investigate the role of social media in pediatric communication, an inferential statistical analysis was conducted to assess the presence of statistically significant associations between the main variables explored, setting the significance level at *p* < 0.05.

First, the association between the frequency with which parents follow pediatricians on social networks and the tendency to change behaviors or decisions regarding their children’s health was studied. To ensure a consistent comparison, the categories “Do not use social media,” as not exposed to the independent variable, and “I don’t remember,” not interpretable for dichotomous classification purposes, were excluded from the inferential analysis. The category “I do not follow pediatrician on social media” was maintained as an internal comparison group because these parents were still social media users and could be indirectly exposed to pediatric content through algorithmic recommendations, reposted material, or other digital sources. Fisher’s exact test confirms a statistically significant association between the two variables (*p* < 0.0001), indicating that exposure to pediatric information content may be associated with a real influence on child health management (Figure S1, Supplementary Materials).

The association between the frequency with which parents follow pediatricians on social media and the perceived usefulness of medical information found online was then analyzed. Only participants who use social media and who, to varying degrees, follow or do not follow pediatricians online were included in the inferential analysis. The “I do not use social media” group was excluded from the analysis because it had no exposure to the variable of interest. Fisher’s exact test showed a statistically significant association between the two variables (*p* < 0.0001), suggesting that greater exposure to online pediatric content is related to a higher perception of its usefulness. The percentage distribution of the three categories of usefulness for each level of following pediatricians on social media is shown in Figure S2 (Supplementary Materials).

To assess whether the frequency with which parents follow pediatricians on social media influences their tendency to discuss such content with their doctor, Fisher’s exact test was conducted, which did not show a statistically significant association between the two variables (*p* = 0.4644), indicating that the likelihood of discussing digital content with their doctor does not vary according to the frequency with which they follow pediatricians on social media.

The association between parental education level and the perceived usefulness of medical information on social media was also analyzed. The three levels of education (middle school diploma, high school diploma, university degree) and the three levels of perceived usefulness (“very useful,” “not very useful,” “not useful at all”) were included. However, Fisher’s exact test did not reveal a statistically significant association between the two variables, suggesting that the perceived usefulness of digital health content is evenly distributed across different levels of education (*p* = 0.9343).

The relationship between the perceived usefulness of medical information found on social media and parents’ tendency to change behaviors or decisions regarding their children’s health was also investigated. The ‘I don’t remember’ category was excluded as it could not be interpreted unambiguously and was not logically comparable with the dichotomous responses (yes, no) representing the actual modes of the dependent variable. Fisher’s exact test showed a statistically significant association (*p* < 0.0001), indicating that in this cross-sectional sample, the perception of high usefulness of information is closely related to a greater likelihood of changing one’s behavior (Figure S3, Supplementary Materials).

The association between changes in parental behavior following exposure to pediatric content on social media and the tendency to discuss it with the treating physician was then analyzed. Again, the group of subjects who answered “I don’t remember” to the question about behavioral change was excluded from the analysis, as it was not logically comparable with the dichotomous categories. Fisher’s exact test did not show a statistically significant association between the two variables (*p* = 0.0865), supporting the finding that behavioral change is not associated with a greater propensity to discuss digital content with the treating physician.

Finally, the inferential analysis was further extended to assess whether certain sociodemographic variables could be associated with the use and evaluation of online pediatric information. In particular, the age of the parent and family composition (number of children) were considered in relation to the perceived usefulness of health information on social media and the frequency of pediatrician follow-ups. For the purposes of inferential analysis, the original age groups were merged into two groups (≤35 years and >35 years) in order to reduce the number of cells with low or zero frequencies and ensure the statistical robustness of Fisher’s exact test.

With regard to the association between parental age and the perceived usefulness of pediatric information on social media, Fisher’s exact test did not reveal any statistically significant association (*p* = 0.0996), supporting an overall similar distribution of usefulness ratings across the different age groups considered. On the other hand, a statistically significant association was demonstrated between parental age and following pediatricians on social media (Fisher’s exact test, *p* = 0.0038). In particular, younger parents are more frequently exposed to pediatric content online than parents over the age of 35, among whom there is a higher proportion of non-exposure or non-use of social media. The modes of exposure to pediatric content on social media were reclassified by combining positive responses (“frequently” and “occasionally”) into a single exposure category to allow for a statistically reliable comparison. The trend of this relationship is shown in Figure S4 (Supplementary Materials).

The association between parents’ age and the tendency to report changes in behavior or decisions related to their children’s health following exposure to pediatric content on social media was also analyzed, but no significant associations were found (Fisher’s exact test *p* = 0.0700).

With regard to the number of children, the analysis of the association with the perceived usefulness of pediatric information on social media showed a homogeneous distribution of responses, supporting the absence of significant associations (Fisher’s exact test *p* = 0.8114). The analysis of the association between the number of children and changes in parenting behaviors also showed no significant associations using Fisher’s exact test (*p* = 0.6855), as did the analysis of the association between the number of children and exposure to pediatricians on social media (Fisher’s exact test with *p* = 0.3492). Again, responses regarding frequency of exposure were divided into three categories: no follow-up with pediatricians, no use of social media, and exposure to pediatricians on social media.

#### 3.5.2. Multivariable Logistic Regression Analysis

To further investigate independent predictors of parental behavioral change, a multivariable logistic regression model was performed including frequency of following pediatricians on social media, perceived usefulness of medical information found on social media, parental educational level and parental age. The outcome variable was reported behavioral change regarding the child’s health (yes = 1; no = 0), while the “I don’t remember” category was excluded from the analysis. In line with the methodological approach adopted for the inferential analyses, participants who reported not using social media were excluded when follow-up of pediatricians on social media was considered as the main predictor.

**Figure 6:**
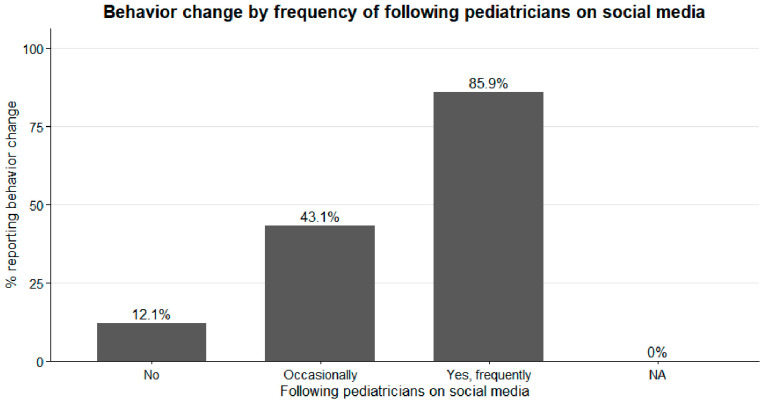
Percentage of participants reporting behavioral change according to the frequency of following pediatricians on social media.

Higher frequency of following pediatricians on social media remained significantly associated with reported behavioral change (OR 3.945; 95% CI: 2.33–6.82; *p* < 0.001), indicating that each increase in exposure to pediatric content was associated with a markedly higher likelihood of modifying parenting behaviors. A clear dose–response pattern was also observed, with the proportion of parents reporting behavioral changes progressively increasing according to the frequency of following pediatricians on social media (Figure 6).

Perceived usefulness of medical information was the strongest predictor (OR 9.967; 95% CI: 4.61–22.64; *p* < 0.001), showing that parents who considered online pediatric information more useful were substantially more likely to report behavioral changes.

**Figure 7:**
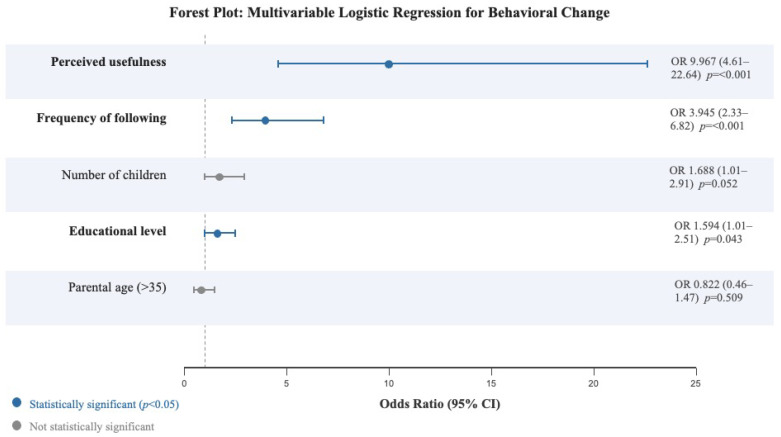
Forest plot showing odds ratios (ORs) and 95% confidence intervals from the multivariable logistic regression model for reported behavioral change.

Higher parental educational level was also independently associated with behavioral change (OR 1.594;95% CI: 1.01–2.51; *p* = 0.042), whereas parental age did not show a statistically significant association (*p* = 0.509). These findings support the hypothesis that both greater exposure to pediatricians’ communication on social media and a higher perceived usefulness of online health information play a major role in shaping parental health-related decisions, independently of age and educational level. Figure 7 summarizes the magnitude and direction of the associations identified in the multivariable logistic regression model.

The overall model fit was satisfactory, with a Nagelkerke R^2^ of 0.457, indicating that the model explains approximately 45.7% of the variance in reported behavioral change.

## 4. Discussion

The results of this study suggest that, within our sample, social media may represent a relevant source of pediatric health information for parents and may be associated with behaviors and decisions related to their children’s health. A large proportion of participants reported following one or more pediatricians on social media regularly (74.2%). However, these findings should be interpreted considering the sampling strategy, as the questionnaire was disseminated through social media and messaging platforms, which may have introduced a degree of self-selection bias, although the inclusion of WhatsApp parent groups, primarily used as a direct messaging tool rather than a social media platform, may have helped reduce, though not eliminate, this concern to some extent.

In Europe, approximately 90% of parents report using digital media to obtain health information [23]. In the United States, almost all parents use social media (96%), although only 68% report using it specifically for health-related purposes [24]. Similarly, Argentinian data indicate widespread use of digital resources for health and parenting topics (70.6%), but show that the majority of parents do not follow pediatricians on social media, with only 34.3% following one to five and just 5% following six or more [16]. An Australian study further reports a high use of social media alone (82.2%) for pediatric health information; however, this analysis focused on generalist platforms, where most content is created and shared by parents [25].

The sociodemographic profile of the sample in this study, characterized mainly by adult parents with a medium-high level of education, suggests a context of good familiarity with digital resources and a potentially greater focus on issues related to the health and well-being of children. This is in line with some data in the literature, showing an association between higher levels of education and greater eHealth literacy [26], i.e., a more developed ability to find, understand, and critically evaluate online health information. In addition, inferential analysis revealed a statistically significant association between parental age and following pediatricians on social media, with younger parents more frequently exposed to online pediatric content, suggesting that age may be a significant determinant in the adoption of social media as a health information channel, likely reflecting generational differences in digital habits.

Social media has progressively established itself as a new dimension of healthcare communication [4]. Over the years, this evidence has become increasingly robust, including in the field of pediatrics, where pediatricians who share information are taking on a new role, no longer simply disseminators but a true digital extension of the healthcare system [5,6].

The areas of greatest interest appear to be fever and common illnesses, growth stages, weaning and nutrition, and sleep, areas that involve everyday parenting, consistent with what has emerged in the literature, where parents frequently use digital media to find information on common issues related to the health and growth of their children [16]. The presence of clear, practical, and reassuring guidance in these areas can play a decisive role in guiding parental choices, as parents tend to experience uncertainty around nutrition and daily routine and are therefore more receptive to accessible information, facilitating its translation into concrete behavioral changes [18].

A particularly relevant finding concerns the behavioral impact of pediatric communication on social media: almost two-thirds of parents reported having modified at least one decision or behavior related to their child’s health after exposure to online pediatric content, with statistically significant associations between reported behavioral changes and both frequency of following pediatricians and perceived usefulness of online information.

However, this finding should not be considered self-evident. In behavioral science it is well established that exposure to health information or changes in attitudes and intentions do not necessarily translate into actual behavior change, a phenomenon often described as the “intention–behavior gap” [24,25]. Notably, the multivariable logistic regression analysis further strengthened these findings, showing that both the frequency of following pediatricians on social media and the perceived usefulness of online medical information remained independently associated with reported behavioral change after adjustment for parental age and educational level. In particular, perceived usefulness emerged as the strongest predictor, suggesting that the subjective appraisal of online pediatric information may play a key mediating role in the translation of digital exposure into self-reported behavioral modifications.

In terms of perceived usefulness, more than 80% of participants rated the information found on social media as very useful, a proportion higher than that reported in the recent paper by Urman et al. [16], which reports a more moderate perception of usefulness. Similar findings were reported in a study conducted in Switzerland by Jaks et al. [23], which highlighted that, although parents recognize digital media as useful sources of health information, a cautious and critical attitude toward online content remains prevalent. This relatively high perceived usefulness of health information found on social media should be interpreted in light of the characteristics of our sample, which appears to be widely exposed to informational content produced by pediatricians on social media. Indeed, over 90% of participants say they follow one or more pediatricians who share information online, with varying frequency.

At the same time, a significant association was found between frequency of follow-up and perceived usefulness of information (*p* < 0.0001): parents who follow pediatricians more closely were more likely to rate the information as very useful. However, these relationships should be interpreted cautiously, as they can reflect associations observed within a cross-sectional design rather than causal pathways. These results support the findings of the scoping review by Kaňková et al. [7], where the effectiveness of messages conveyed by social media influencers was found to depend largely on the audience’s perception of credibility, relevance, and usefulness. In digital communication contexts, these factors are frequently described as mediating elements between exposure to content and potential engagement or behavioral responses [4,14,16]. Similarly, Bozzola et al. [5] show that the effectiveness of communication on social media in the paediatric field is closely associated with the perceived practicality and immediate usefulness of the information provided.

The inferential analysis does not reveal a statistically significant association between the perceived usefulness of health information on social media and the parents’ educational level, although parents with a low level of education are underrepresented in the present sample. Therefore, the perception of usefulness may depend less on critical skills and more on cross-cutting elements such as clarity of communication, usefulness, comprehensibility of the message, and perception of the authority of the source. These dimensions, shared by parents with different educational levels, could mitigate any differences in the subjective assessment of usefulness. This interpretation is consistent with the findings of Milanti et al. [26], according to whom eHealth literacy is a multifactorial construct influenced not only by educational attainment but also by digital, motivational and contextual skills, and with Urman et al. [16], who show that the use of digital media for health and parenting information is now widespread across different educational levels.

Trust also appeared to be partially influenced by personal familiarity with the pediatrician and, to a lesser extent, by the sharing of elements of private life. This finding may reflect the relational nature of social media communication, where narrative and personal elements can increase perceived accessibility and relational closeness, fulfilling relational and emotional needs for parents, including reassurance, peer comparison, emotional support, and the possibility of accessing experiences shared by other caregivers, as highlighted by Frey et al. [25].

However, credibility should remain primarily grounded in professional competence and evidence-based communication, as highlighted by La Bella et al. [6] and AAP recommendations [10].

Parental concerns regarding pediatricians’ presence on social media, including the risk of replacing clinical visits, misunderstandings, confusion due to the presence of discordant opinions among pediatricians, and dissemination of overly generic or non-personalized information, are fully consistent with what has been highlighted in the scientific literature since the first studies on the subject [4]. In this regard, several studies have highlighted how misinformation can generate distorted perceptions of competence and how digital environments tend to amplify cognitive biases, promoting misinterpretations and potentially inappropriate healthcare decisions [8,9,14]. These dynamics are further explained by the echo chamber phenomenon, whereby digital environments favor ideologically homogeneous communities with selective exposure to content, contributing to the amplification of misinformation and undermining trust in authoritative sources [8,9].

A further dimension that the present study did not explicitly address concerns the role of peer-to-peer digital environments, such as parenting blogs, online forums, and parent-led social media groups, as an additional and potentially competing source of pediatric health information. Evidence suggests that peer-generated content, influencer communication, and short-video platforms such as TikTok (TikTok Inc., Los Angeles, CA, USA) too often represent additional sources of non-evidence-based pediatric health information, with highly variable quality and a greater prevalence of misinformation than accurate, evidence-based guidance [17,20,21,22].

These findings reinforce the importance of a strong, recognizable, and professionally grounded presence of pediatricians on social media, as a counterweight to the peer-generated information ecosystem in which parents are increasingly immersed. In this context, informative content on social media sometimes can take the form of a sort of “advance or parallel consultation”, through which parents begin to form an opinion before the clinical appointment, build expectations and, in some cases, make independent decisions, with the risk that digital communication may be interpreted as a substitute for traditional medical consultation rather than as a complementary tool [6,10]. However, the most recent data do not seem to support this concern: Urman et al. [16] report that most parents would not replace visits to the pediatrician with online consultations, although 36% often supplement the information received during the visit with details found in digital media, a finding further confirmed by Jaks et al. [23].

At the same time, the strong consensus in favor of introducing regulations on pediatric disclosure on social media (72.6%) suggests that parental awareness of these risks translates into an active demand for institutional protection and regulatory clarity, going beyond mere recognition of potential concerns. The homogeneous distribution of risk perception across educational levels may reflect a cross-sectional diffusion of digital health literacy skills among parents regardless of formal education, consistent with the multifactorial nature of eHealth literacy [26], and indicative of an overall ambivalent but informed attitude toward pediatric communication on social media.

Particular attention should be paid to the lack of integration between online information and the doctor-patient relationship, despite the widespread use of social media. In fact, in the overall sample, only 104 parents (22.9%) reported having discussed pediatric content seen on social media with their doctor. However, considering only parents who directly follow pediatricians on social media, the percentage stands at 23.8% (100 parents), confirming that the majority of parents tend not to systematically integrate such content into clinical discussions with their trusted doctor. Consistently, the inferential statistical analysis did not show any significant relationship between the frequency of following pediatricians and the tendency to discuss such content with one’s doctor, nor between the change in behavior and the discussion with the pediatrician. This suggests that information from social media often remains confined to an autonomous sphere, not integrated into the clinical relationship, consistent with previous literature [4,24]. However, the poor integration between social information and clinical dialogue observed cannot be interpreted solely as a choice made by parents, but also as the result of a structural difficulty in rethinking the doctor-parent relationship in a digital context. In fact, although a study [27] shows that pediatricians predominantly adopt patient-centered communication strategies towards parents who access health information online, with frequent use of co-constructive methods based on the recognition and contextualization of digital sources, the authors emphasize the simultaneous, albeit minority, presence of directive and less dialogical approaches [27]. The data from our study describe a situation that is less favorable to co-constructive dialogue, highlighting that among parents who actually discussed the content found on social media with their pediatrician, only 47.1% reported a positive or interested response, while a significant proportion described doubtful or cautious reactions (24%), questioning the reliability of online sources (16.3%), or an annoyed and uncooperative attitude (12.5%). These data can be interpreted in light of pediatricians’ stated need for greater support in adapting to the “informed parent,” who may be misled by online health information and critical of pediatricians’ clinical practice [26]. This supports the need to train pediatricians to improve their communication skills in the digital age, as well as recognizing doctors active on social media as a distinct category of digital healthcare professional, differentiated from generalist influencers by their professional qualifications, ethical responsibilities and evidence-based approach, alongside the adoption of clear and rigorous regulations [7].

However, the need to promote more participatory communication models is emerging in an attempt to mitigate the interpretative risks associated with the autonomous consumption of digital information, particularly given that pediatric communication on social media still largely follows a one-way dissemination model that limits opportunities for structured dialogue and shared decision-making with parents [5]. Some authors have pointed out that unmediated exposure can distort the perception of skills and lead to inappropriate health decisions [14] and that low interaction with professionals increases vulnerability to misinformation [9]. Furthermore, co-constructive approaches are more effective than directive or dissuasive responses [14]. The need for greater integration between digital media and healthcare providers is confirmed by the study by Frey et al. [25], in which the majority of digital media users (67%) stated that they would like to receive recommendations on reliable digital sources from their pediatrician.

## 5. Strengths and Limitations

This study has some methodological limitations that should be considered when interpreting the data with regard to sampling, which was based mainly, although not exclusively, on dissemination via social media and digital channels, there is a potential bias of self-selection. In other words, participants are likely to be more interested in digital communication than the general population of parents, with possible repercussions on both the frequency of social media use for informational purposes and the perception of the usefulness of informative content. It should also be noted that the sociodemographic composition of the sample, characterized by a low representation of individuals with lower levels of education, may have influenced the associations between educational level, perceived usefulness, and perception of risks. In addition, the absence of validated measurements of participants’ eHealth literacy may limit the ability to fully interpret the relationship between educational level and perceived usefulness of online health information.

Another limitation concerns the self-reported nature of the data collected, which may be subject to memory bias and social desirability bias, resulting in inaccurate data or responses dictated by greater social acceptability, especially with regard to changes in parental behavior and discussions with the attending physician. In addition, the questionnaire was not formally piloted prior to dissemination, nor was a structured assessment of content validity or comprehensibility conducted. This may have introduced variability in the interpretation of individual items by participants.

Furthermore, in the descriptive phase, some inconsistencies emerged between the responses provided by participants, particularly between statements relating to following pediatricians on social media and the subsequent statement of having discussed content shared by pediatricians on social media with their treating physician. The presence of affirmative responses among parents who reported not following pediatricians on social media may reflect an interpretative ambiguity rather than a true inconsistency. Participants may have been exposed to pediatric content indirectly through algorithmic recommendations, reposted material, parenting groups, or content shared by other users, without actively following pediatricians’ profiles. Therefore, the concept of “content shared by a pediatrician” may have been interpreted more broadly than intended. This interpretative ambiguity made it necessary to exclude some categories from the inferential analyses to ensure logical consistency, with a consequent reduction in the possibility of generalizing statistical inferences.

Finally, the cross-sectional nature of the study design does not allow for the establishment of causal relationships between exposure to pediatric content on social media and changes in parenting behaviors. Although statistically significant associations were found, it is not possible to determine whether exposure to digital content actually led to behavioral change or whether, on the contrary, parents who are more inclined to change are also more inclined to actively seek out and follow pediatric content online. Furthermore, the absence of longitudinal follow-up does not allow assessment of whether the patterns observed remain stable over time. Future studies adopting a longitudinal design with repeated measurements would allow verification of the consistency of parental perceptions and behaviors related to pediatric communication on social media. Similarly, the poor integration of digital content into the doctor-parent dialogue cannot be attributed solely to the initiative of families, as the questionnaire did not explore any perceived barriers such as fear of judgment, limited time during the visit, and the attitudes of the professional.

The specific focus on pediatricians operating on social media represents an area that has been little explored in the international literature, which has traditionally concentrated on the broader use of digital media for health information searches.

The detailed structure of the questionnaire also allowed for an integrated analysis of perceptions, behaviors, and consequences reported by parents, including comparison with the attending physician, an aspect rarely explored in previous studies, allowing for a multidimensional assessment of parents’ digital behaviors.

The inclusion of an inferential analysis broadens the interpretative scope of the results, allowing the identification of significant patterns of association between exposure to pediatric content, perceived usefulness, and behavioral change.

Finally, the results have direct practical relevance for pediatric communication strategies, offering concrete insights for physicians and healthcare institutions aiming to improve the digital engagement of families.

## 6. Conclusions

Pediatric outreach on social media in the Italian context now appears to be an increasingly relevant component in the parental information process. Our findings suggest that communication by pediatricians through digital platforms may contribute to improving access to health information, prevention messages, and parental support, supporting its potential role as a complementary tool in pediatric health promotion.

Most participants regularly follow pediatricians on social media and consider the informative content to be reliable, relevant, and capable of providing immediate support for daily parenting needs. These data suggest the presence of a specific digital space, attributable to the professional communication of pediatricians on social media, which differs from the use of the Internet or social media not managed by healthcare professionals. In this space, the figure of the pediatrician takes on a public and informative dimension, suggesting a distinct model of information use, perceived as more reliable and immediately applicable.

Despite this, pediatricians’ digital communication seems to be predominantly a one-way information environment, closer to a top-down model than to dialogical-participatory communication, in line with more traditional communication models.

Parents’ limited tendency to discuss such content with their doctor suggests that digital information tends to remain confined to the personal sphere, without being fully integrated into the doctor-patient relationship. This disconnect appears particularly relevant in light of the high perceived usefulness: online dissemination is considered effective and reassuring, but does not yet translate into a tool for shared clinical discussion. This gap is identified as a possible area for intervention, both in terms of the communication strategies of pediatricians who share information, who could promote greater bidirectionality, and in terms of daily clinical practice, where doctors could explicitly invite critical sharing of digital content.

Ultimately, pediatricians engaged in social media communication today represent a promising opportunity to broaden the reach of evidence-based information and support parents in a digital environment saturated with non-professional content. In order for this potential to translate into a real improvement in the care relationship, it will be necessary to develop specific communication skills, ethical guidelines, and pathways that facilitate the integration of online information and clinical practice, including by beginning to define pediatricians active on social media as a distinct category of digital health communicators, differentiated from generalist influencers by their professional qualifications, ethical responsibilities, and evidence-based approach. Only in this way can pediatric communication on social media evolve from a simple flow of information to a tool for participation, alliance, and shared awareness.

## Figures and Tables

**Table 1:** Questionnaire structure and item type.

Section	Main Content/Questions Included	Number of Item	Response Type
1. Socio-demographic data	Parent’s age; Educational qualification; Number of children; Age of the child in question.	4	Polytomous/multiple
2. General use of social media and perception of pediatric communication	Do you follow one or more pediatricians on social media? How useful do you find the medical information you find on social media? Which of these topics do you search for most frequently online (you can select more than one answer)? How do you prefer to receive medical information on social media? Do you think that communication on social media by paediatricians can help improve information, prevention and support for parents? Would you trust online advice more if you knew the paediatrician who shared it personally? How much does the sharing of personal content (family photos, daily life) on a paediatrician’s social media page affect your trust in them? Have you ever discussed content shared by a paediatrician on social media with your doctor? If so, what was your doctor’s reaction?	9	5-point Likert scale/multiple choice/dichotomous
3. Impact on parenting behaviors	Have you ever changed your behavior based on information obtained from social media? In what area? (weaning/feeding, breastfeeding, sleep, fever and common illnesses, vaccinations or medications, other)	2	Polytomous/multiple
4. Risks and regulation	What risks or negative aspects do you associate with pediatricians’ presence on social media? Would you feel more secure if there were regulations regarding pediatric disclosure on social media?	2	Polytomous/multiple
Total		17 items	

Note: The Likert scale questions were scored from 1 to 5, where 1 indicated the minimum level of agreement or perceived usefulness and 5 the maximum level. Some items used a 3-point scale (1 = negative effect; 2 = neutral; 3 = positive). Multiple-choice questions allowed the selection of multiple options from a predefined list, while dichotomous and polychotomous questions had yes/no and yes/no/do not remember answers.

## Data Availability

The original contributions presented in this study are included in the article. Further inquiries can be directed to the corresponding author.

## Supplementary Materials

The following supporting information can be downloaded at: https://www.mdpi.com/article/10.3390/children13070923/s1, Figure S1. Percentage Distribution of Behavioral-Change Responses Across Levels of Parental Engagement with Pediatricians on Social Media; Figure S2. Distribution of Perceived Usefulness of Medical Information by Parents’ Engagement with Pediatricians on Social Media; Figure S3. Association Between Perceived Usefulness of Medical Information Found on Social Media and Reported Changes in Parental Behavior; Figure S4. Distribution of Exposure to Pediatricians’ Content on Social Media According to Parents’ Age Group; STROBE Statement—Checklist of items that should be included in reports of cross-sectional studies.
