# Supplementary material for: Parents’ Perception of Pediatricians on Social Media: The Emerging Role of Pediatric Health Communicator
Source: Children (Basel). 2026 Jul 13;13(7):923. doi: 10.3390/children13070923 (PMC13407234; doi:10.3390/children13070923)
Supplement: Supplementary file 1 [file children-13-00923-s001.zip › children-4355621-supplementary.pdf]

Supplementary Materials

Figure S1. Percentage Distribution of Behavioral-Change Responses Across Levels of Parental Engagement with Pediatricians on Social Media.

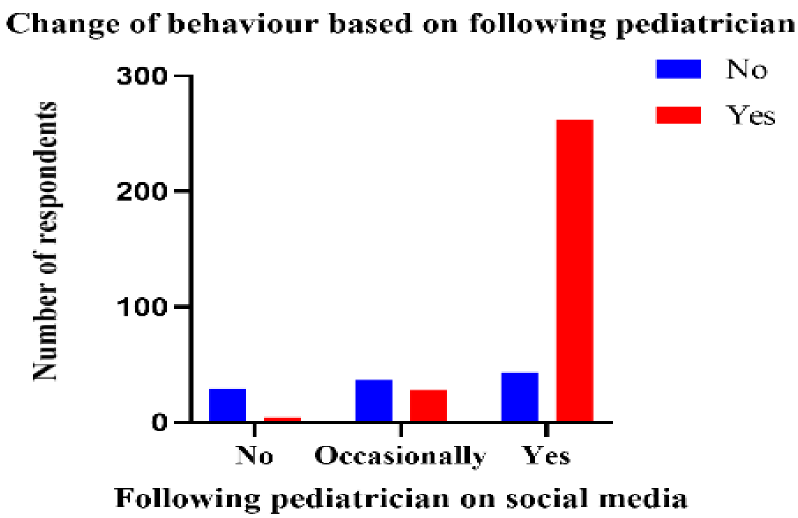

Figure S2. Distribution of Perceived Usefulness of Medical Information by Parents' Engagement with Pediatricians on Social Media.

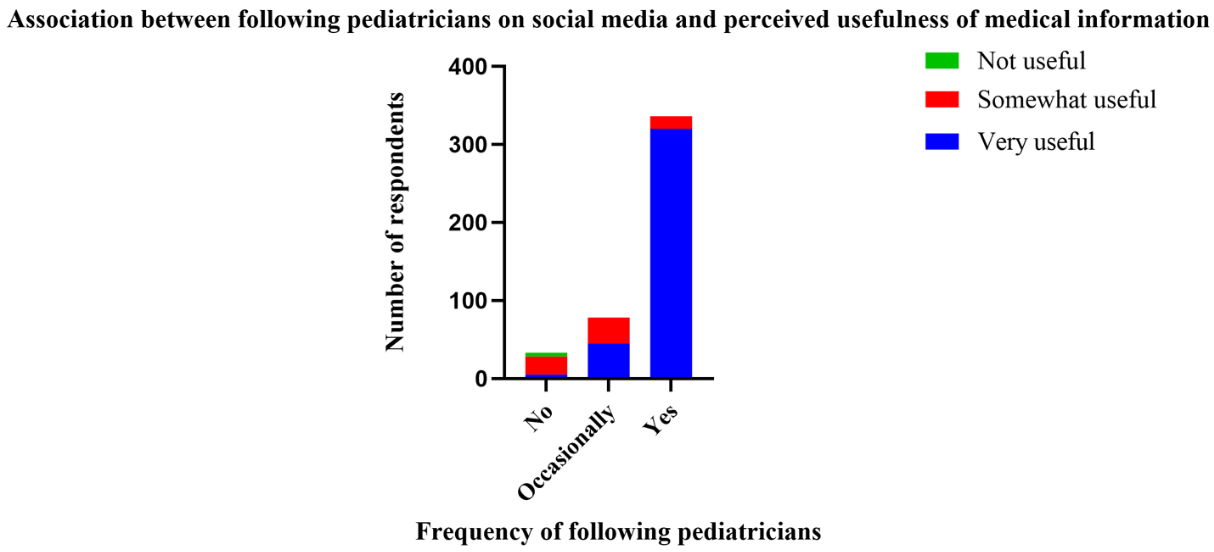

Figure S3. Association Between Perceived Usefulness of Medical Information Found on Social Media and Reported Changes in Parental Behaviour.

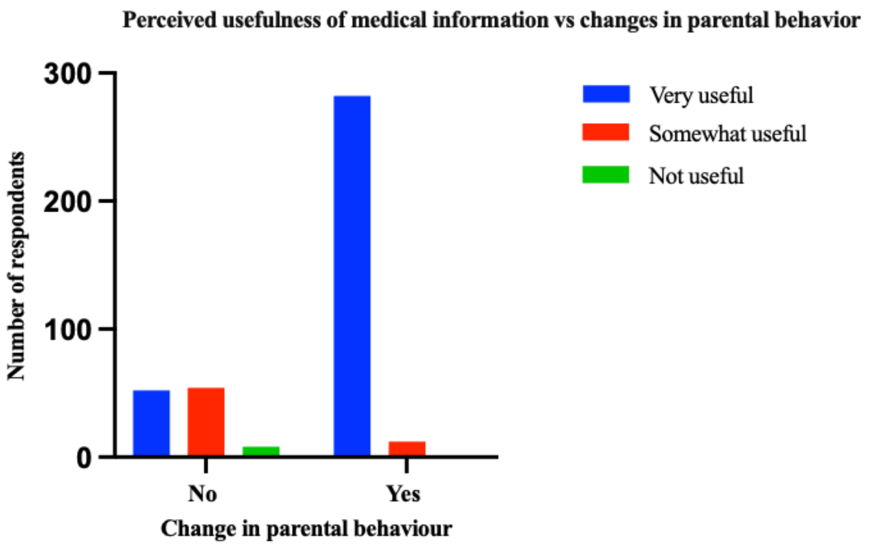

Figure S4. Distribution of Exposure to Pediatricians' Content on Social Media According to Parents' Age Group.

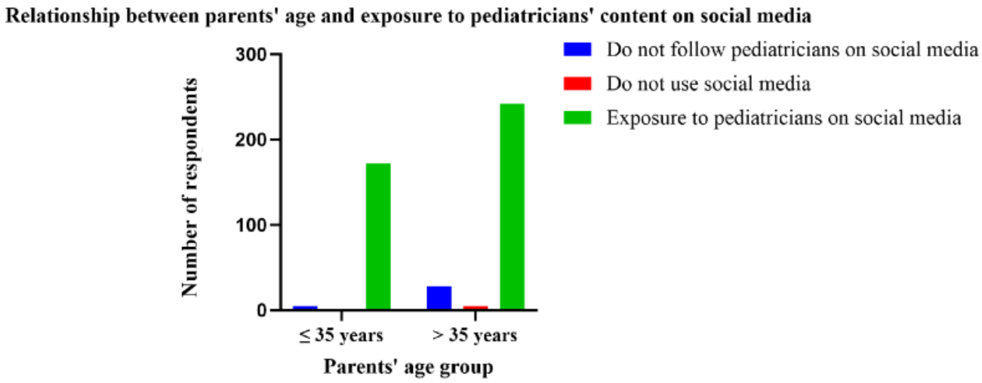

STROBE Statement—Checklist of items that should be included in reports of ***cross-sectional studies***

	Item No	Recommendation	Page No / Section

**Title and abstract**	1	(a) Indicate the study's design with a commonly used term in the title or the abstract	Abstract (Methods): 'cross-sectional observational study'
		(b) Provide in the abstract an informative and balanced summary of what was done and what was found	Abstract: structured summary (Background, Methods, Results, Conclusions)
**Introduction**			
Background/rationale	2	Explain the scientific background and rationale for the investigation being reported	Introduction, pp. 1–2
Objectives	3	State specific objectives, including any prespecified hypotheses	Introduction, final paragraph, p. 3
**Methods**			
Study design	4	Present key elements of study design early in the paper	Section 2.1, p. 3
Setting	5	Describe the setting, locations, and relevant dates, including periods of recruitment, exposure, follow-up, and data collection	Section 2.1: Italy, June 2025, Instagram and WhatsApp dissemination
Participants	6	(a) Give the eligibility criteria, and the sources and methods of selection of participants	Section 2.2, p. 3: inclusion/exclusion criteria
Variables	7	Clearly define all outcomes, exposures, predictors, potential confounders, and effect modifiers. Give diagnostic criteria, if applicable	Sections 2.3–2.4; Table 1: outcome = behavioral change; predictors = frequency of following, perceived usefulness, age, education
Data sources/measurement	8*	For each variable of interest, give sources of data and details of methods of assessment (measurement). Describe comparability of assessment methods if there is more than one group	Section 2.3, p. 3–4; Table 1: 17-item questionnaire, Likert/dichotomous/multiple-choice items
Bias	9	Describe any efforts to address potential sources of bias	Incomplete questionnaires excluded (Section 2.2); bias acknowledged in Section 5. Regarding duplicate responses, the platform was configured to hide the option to resubmit upon completion; the potential for duplicate responses is acknowledged as a limitation of anonymous online survey methodology (Section 5).
Study size	10	Explain how the study size was arrived at	No formal sample size calculation was performed. The study was designed as an exploratory cross-sectional survey; 453 participants completed the questionnaire (Section 2.2).
Quantitative variables	11	Explain how quantitative variables were handled in the analyses. If applicable, describe which groupings were chosen and why	Section 3.5.1: age dichotomized ≤35/>35 years with rationale; exposure categories merged with rationale

Statistical methods	12	(a) Describe all statistical methods, including those used to control for confounding	Section 2.4: Fisher's exact test; multivariable logistic regression (confounders: age, education)
		(b) Describe any methods used to examine subgroups and interactions	Section 3.5.1: subgroup analyses by age, education, number of children
		(c) Explain how missing data were addressed	Section 2.2: incomplete questionnaires excluded; 'I don't remember' excluded from specific analyses with rationale
		(d) If applicable, describe analytical methods taking account of sampling strategy	Convenience sampling; no weighted analysis required; limitation acknowledged (Section 5)
		(e) Describe any sensitivity analyses	Not performed; not applicable given exploratory design
**Results**			
Participants	13*	(a) Report numbers of individuals at each stage of study	Section 3.1: 453 participants; all confirmed consent; incomplete questionnaires excluded per protocol
		(b) Give reasons for non-participation at each stage	Not collected (anonymous open-access design; not applicable)
		(c) Consider use of a flow diagram	Not included; inclusion/exclusion criteria described in Section 2.2 (not applicable for anonymous online survey)
Descriptive data	14*	(a) Give characteristics of study participants (eg demographic, clinical, social) and information on exposures and potential confounders	Section 3.1, p. 5: age, education, family composition reported for all 453 participants
		(b) Indicate number of participants with missing data for each variable of interest	All items were mandatory for questionnaire progression; incomplete questionnaires were excluded per protocol (Section 2.2), resulting in no missing data for the primary variables of interest. Multiple-response items were clearly indicated within the questionnaire.
Outcome data	15*	Report numbers of outcome events or summary measures	Sections 3.2–3.4 and Figures 1–11: 65% behavioral change; 74.2% follow pediatricians; 81.7% very useful; 22.9% discussed with doctor
Main results	16	(a) Give unadjusted estimates and, if applicable, confounder-adjusted estimates and their precision (eg, 95% confidence interval). Make clear which confounders were adjusted for and why they were included	Section 3.5.2: OR, 95% CI and p-values reported for all predictors.

		(b) Report category boundaries when continuous variables were categorized	Section 3.5.1: age ≤35 vs >35 years; exposure frequency categories described with rationale
		(c) If relevant, consider translating estimates of relative risk into absolute risk for a meaningful time period	Not applicable (cross-sectional survey, no incidence data)
Other analyses	17	Report other analyses done—eg analyses of subgroups and interactions, and sensitivity analyses	Section 3.5.1: all bivariate subgroup analyses reported; no sensitivity analyses performed
**Discussion**			
Key results	18	Summarise key results with reference to study objectives	Section 4, opening paragraph, p. 11
Limitations	19	Discuss limitations of the study, taking into account sources of potential bias or imprecision. Discuss both direction and magnitude of any potential bias	Section 5, pp. 13–14: self-selection, self-report, social desirability, underrepresentation of low education, cross-sectional design, no validated eHealth literacy measure, no pilot test (added per Reviewer 2)
Interpretation	20	Give a cautious overall interpretation of results considering objectives, limitations, multiplicity of analyses, results from similar studies, and other relevant evidence	Section 4, pp. 11–13: contextualised with literature; causal language avoided; cross-sectional nature noted
Generalisability	21	Discuss the generalisability (external validity) of the study results	Section 5 and Section 6 Conclusions: self-selection bias and sample characteristics limit generalisability; explicitly acknowledged
**Other information**			
Funding	22	Give the source of funding and the role of the funders for the present study and, if applicable, for the original study on which the present article is based	Funding section: 'This research received no external funding.'

**Give information separately for exposed and unexposed groups.*

Note: An Explanation and Elaboration article discusses each checklist item and gives methodological background and published examples of transparent reporting. The STROBE checklist is best used in conjunction with this article (freely available on the Web sites of PLoS Medicine at http://www.plosmedicine.org/, Annals of Internal Medicine at http://www.annals.org/, and Epidemiology at http://www.epidem.com/). Information on the STROBE Initiative is available at www.strobe-statement.org.
